# Arachnoid granulations are lymphatic conduits that communicate with bone marrow and dura-arachnoid stroma

**DOI:** 10.1084/jem.20220618

**Published:** 2022-12-05

**Authors:** Trishna Shah, Sue E. Leurgans, Rashi I. Mehta, Jingyun Yang, Chad A. Galloway, Karen L. de Mesy Bentley, Julie A. Schneider, Rupal I. Mehta

**Affiliations:** 1 Rush Alzheimer’s Disease Center, Rush University Medical Center, Chicago, IL; 2 Department of Neurological Sciences, Rush University Medical Center, Chicago, IL; 3 Department of Neuroradiology, Rockefeller Neuroscience Institute, West Virginia University, Morgantown, WV; 4 Department of Neuroscience, Rockefeller Neuroscience Institute, West Virginia University, Morgantown, WV; 5 Department of Pathology, University of Rochester Medical Center, Rochester, NY; 6 Department of Pathology, Rush University Medical Center, Chicago, IL

## Abstract

Arachnoid granulations (AG) are poorly investigated. Historical reports suggest that they regulate brain volume by passively transporting cerebrospinal fluid (CSF) into dural venous sinuses. Here, we studied the microstructure of cerebral AG in humans with the aim of understanding their roles in physiology. We discovered marked variations in AG size, lobation, location, content, and degree of surface encapsulation. High-resolution microscopy shows that AG consist of outer capsule and inner stromal core regions. The fine and porous framework suggests uncharacterized functions of AG in mechanical CSF filtration. Moreover, internal cytokine and immune cell enrichment imply unexplored neuroimmune properties of these structures that localize to the brain–meningeal lymphatic interface. Dramatic age-associated changes in AG structure are additionally identified. This study depicts for the first time microscopic networks of internal channels that communicate with perisinus spaces, suggesting that AG subserve important functions as transarachnoidal flow passageways. These data raise new theories regarding glymphatic–lymphatic coupling and mechanisms of CSF antigen clearance, homeostasis, and diseases.

## Teaser

Arachnoid granulations are poorly investigated. We show that they harbor immune cells and communicate with perisinus spaces, suggesting that granulations subserve neuroimmune roles and function as transarachnoidal passageways. These data raise new theories regarding glymphatic–lymphatic coupling and mechanisms of diseases.

## Graphical Abstract

**Figure:**
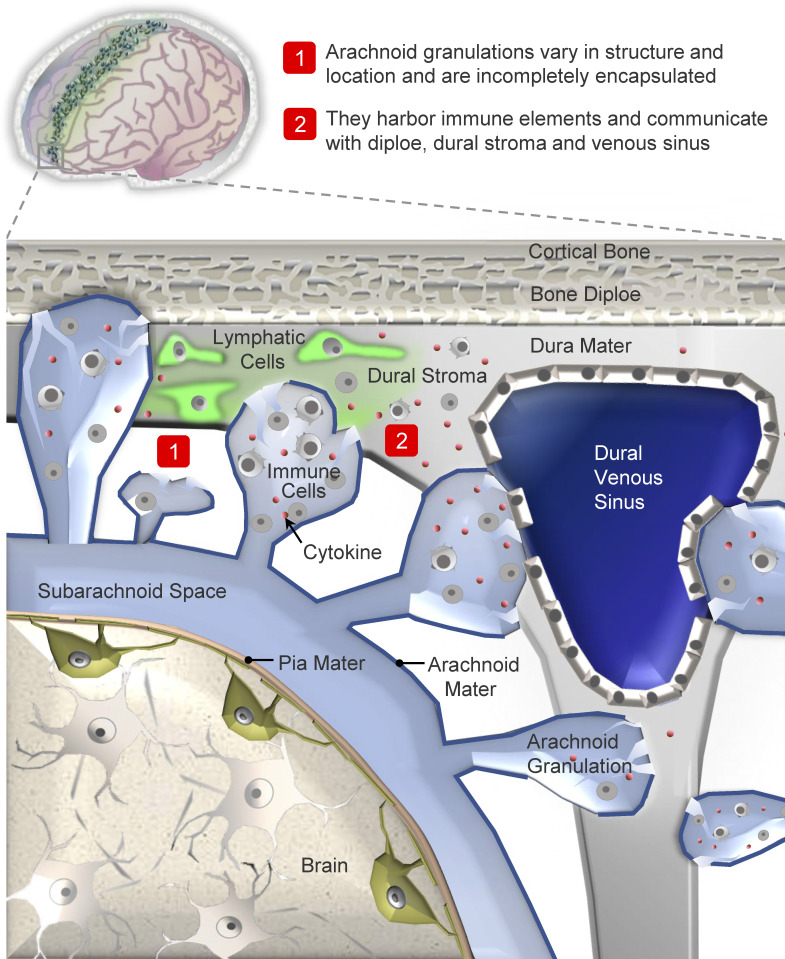

## Introduction

Arachnoid granulations (AG), which are also known as Pacchionian bodies, uniquely localize to the mammalian brain–dura interface and remain minimally investigated across species. These central nervous system (CNS) structures were described as early as the 16th century and were called glandulae congoblatae by Pacchioni, who proposed that they are secretory bodies that have roles in CNS lubrication (Brunori et al., 1993; Pollay, 2010). Conversely, Weed conjectured that AG house internal channels that facilitate egress of cerebrospinal fluid (CSF; Weed, 1914; Weed, 1917). Since these early classical works, AG have generally been regarded as the primary CSF outflow paths responsible for brain volume regulation in humans (Welch and Friedman, 1959; Welch and Friedman, 1960; Wolpow and Schaumburg, 1972). Yet, the precise functions and structure of fluid pathways within AG have remained elusive, and the morphology, cytology, and anatomical relationships of AG have been a matter of debate over centuries, leaving unanswered questions regarding their biological significance at CNS interfaces.

Increasing data suggest that CSF circulation serves critical roles in brain maintenance (Louveau et al., 2015; Da Mesquita et al., 2018a; Mestre et al., 2020). Yet, specific mechanisms and pathways involved in CSF passage within intracranial cavities remain incompletely explained (Keep et al., 2019; Rustenhoven et al., 2021). Historical literature asserts that AG protrude through dura, permitting CSF drainage into dural venous spaces (DVS, i.e., dural venous sinuses, dural veins, and lacunae; Kida et al., 1988; Kida et al., 1993; Welch and Pollay, 1961). Dura and arachnoid mater have been suggested to fuse focally on AG at DVS sites and, consequently, it has been concluded that AG and DVS lumina communicate (Gomez et al., 1974; Nabeshima et al., 1975). However, direct evidence and precise mechanisms and pathways by which CSF is transported within and across AG have not been delineated, and investigation of this tissue is challenging in live humans. Microscopic endothelial-lined gaps, pores, and/or surface crypts have been represented on a few postmortem AG surfaces at DVS interfaces (Gomez et al., 1974; Kida et al., 1988). Some researchers have speculated that internal endothelial-lined nonvascular tubules permit passive fluid flux across AG via open and direct communications (Gomez et al., 1974; Welch and Friedman, 1960; Upton and Weller, 1985), though others purport that AG fluid transmits into DVS via transcellular movement (Kida et al., 1988; Kida et al., 1993; Tripathi, 1977; Weller et al., 2018). To date, the delicate histology of AG has yet to be fully elucidated.

Given the paucity of studies on AG anatomy combined with the lack of systematically acquired histologic evidence in published reports (Jayatilaka., 1965a; Jayatilaka., 1965b; Weller et al., 2018), we here systematically analyzed the anatomy of human AG using a panel of cellular and molecular markers. We show that human AG are comprised of a central collagen framework that is variably encapsulated and only a subset associate with DVS tissues. We also demonstrate that internal collagen forms a stromal meshwork and definitively depict for the first time immune cell enrichment within AG cores. Furthermore, we illustrate that AG domes border nonsinus spaces. Given published in vivo tracer evidence and data presented here, it is inferred that AG fluid permeates freely into perisinus and diploic compartments, and we conclude that AG serve as CSF reservoirs and immune hubs at meningeal interfaces. This study also characterizes prominent age-associated degenerative changes, which may be due to fluid stasis and likely account for anatomic discrepancies in prior reports. In light of recent insights regarding intracerebral fluid movement and the existence of meningeal lymphatic channels in mammalian species (Absinta et al., 2017; Louveau et al., 2015; Da Mesquita et al., 2018a) this work provides a novel conceptual framework for understanding and investigating glymphatic–lymphatic flow and brain antigen processing in mammals and may provide new clues into the neurophysiology and pathogenesis of a range of diseases.

## Results

### AG are heterogeneous in composition

**Figure 1.**
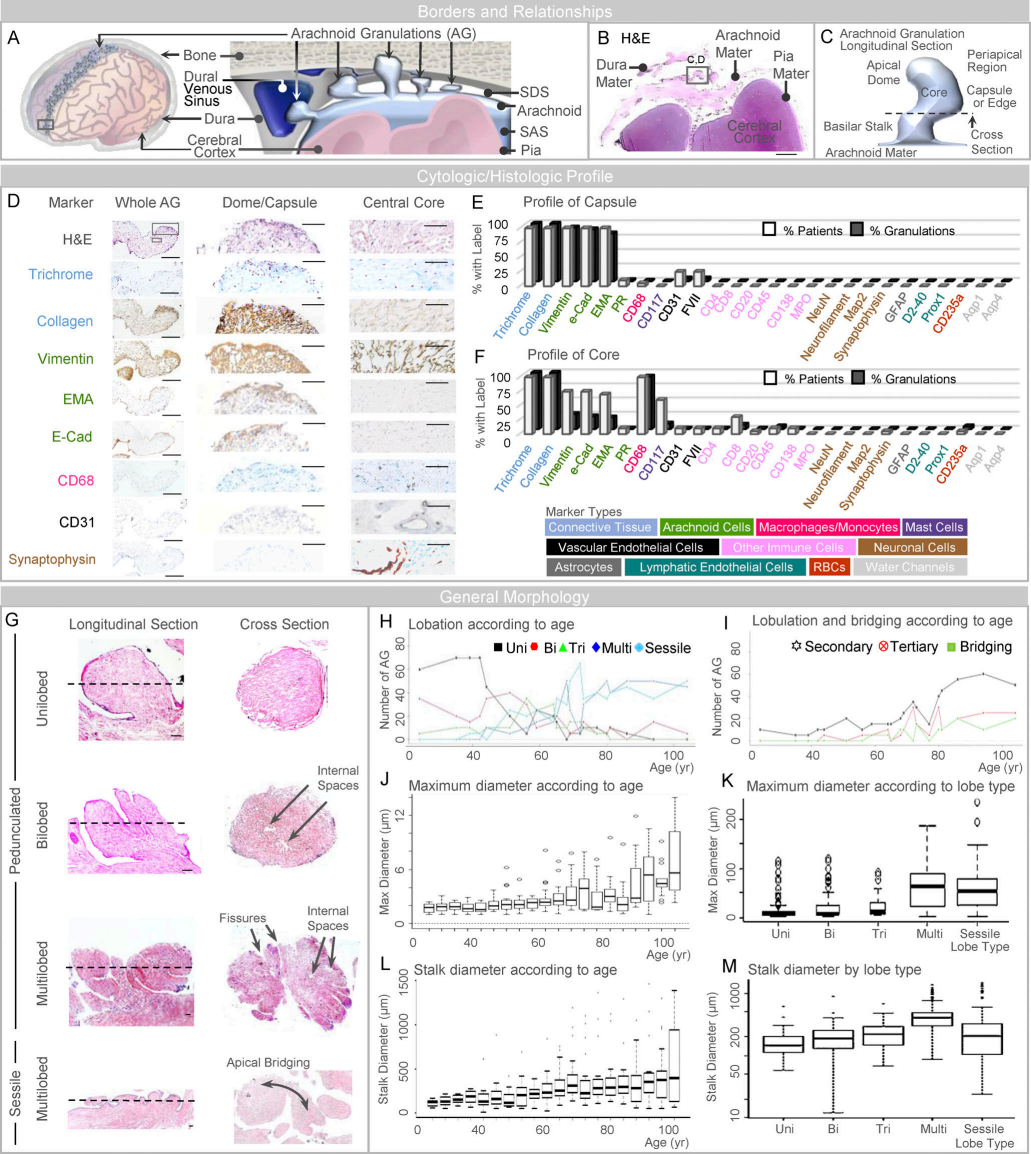
**AG abut dura mater and are heterogeneous in size, form, and composition. (A)** A schematic depicts the general arrangement of AG in the superior parasagittal frontal brain region. **(B)** Similarly, low-power image of an H&E-stained frontal pole section illustrates basic meningeal relationships. **(C)** Schematic depiction of a single granulation, shown in longitudinal orientation, summarizes its general morphology which includes a capsule or edge, core, basilar stalk or neck, and apical dome region. **(D)** Intermediate-power images further illustrate labels of an isolated granulation, including dome capsule and central core regions. **(E and F)** Labeling profiles of each granulation are summarized from all patients at the granulation capsule (or edge) and inner core, respectively, in E and F; cell marker types used for initial screening are shown in the lower panel. **(G)** As illustrated, AG are pedunculated (or polypoid) and unilobed, bilobed, trilobed, or multilobed; or are sessile and plaque-like in morphology; some granulations exhibit secondary and tertiary lobulations with or without apical bridging. **(H and I)** The number of morphologies and the number of lobulated and bridged granulations are summarized by age in H and I, respectively. **(J and K)** Maximum diameter is shown according to age and lobe type in J and K, respectively. **(L and M)** Stalk diameter is shown according to age and lobe type in L and M, respectively. Scale bars: (B) 500 µm; (D [left]) 200 µm; (D [middle and right]) 50 µm; (G) 20 µm. Bi, bilobed; EMA, epithelial membrane antigen; Multi, multilobed; PR, progesterone receptor; Tri, trilobed; Uni, unilobed. Dotted lines in G represent the approximate orientation of cross-section shown on the right-hand side. Data represent findings in 400 frontal pole granulations from 20 decedents and are from more than two independent experiments.

**Figure S1.**
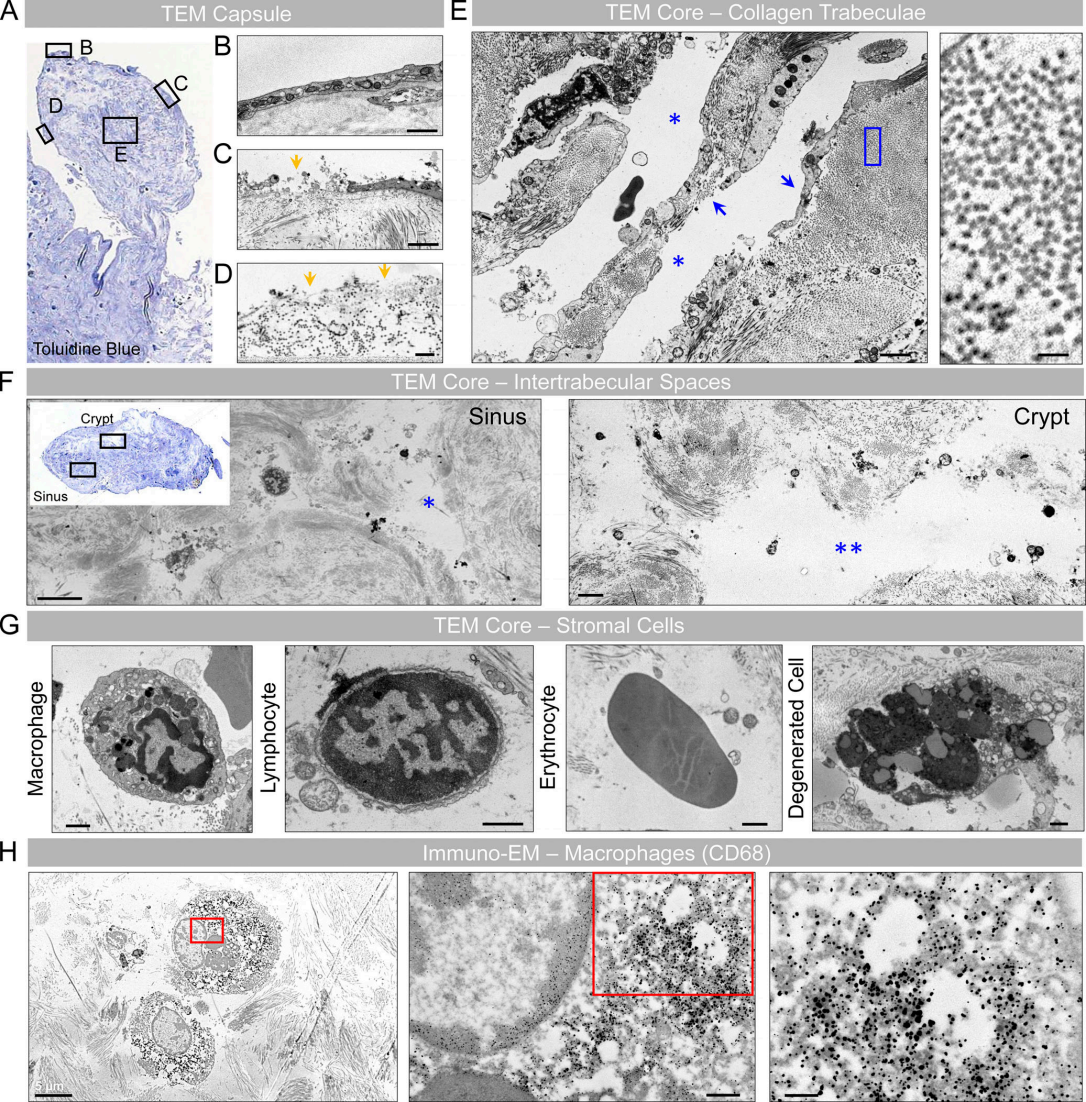
**Ultrastructural images of a unilobed AG depict its surface and internal morphology. (A)** Toluidine blue–stained thin section of a unilobed AG displays the AG stalk and body in longitudinal orientation. **(B–D)** Ultrastructural images of different surface (boxed) regions demonstrate areas of intact (B), partial (C), or no (D) capsular coverage. Note areas of communication between the AG core and periapical region (yellow arrows in C and D). **(E)** The internal core stroma depicts trabeculae that are unlined (left arrow) or lined (right arrow) by fibroblast-like cells, consistent with meningothelium. High-power micrograph image of the blue boxed area is shown in the inset at right and demonstrates a trabeculum comprised of densely packed collagen fibrils. **(F)** A toluidine blue–stained thin section of a different granulation from the same decedent is shown in axial orientation, along with micrographs of the boxed areas. Note sinuses on the left and larger, gaping crypt spaces on the right-hand side. Both the sinus and crypt spaces contain cell debris and rounded mononuclear cells at their margins. **(G)** Enlarged images of sinus and crypt spaces show diverse cell types. **(H)** On an immuno-EM image prepared using a primary antibody directed toward CD68, many rounded stromal cells are confirmed as macrophages; see insets (i.e., enlargements of red boxed areas) at right-hand side that depict cytoplasmic and lysosomal electron-dense label. (A and F [inset]) Toluidine blue–stained thin sections; (B–G) TEM; (H) immuno-electron micrograph. Scale bars: (B, C, and E [left]) 2 µm; (D and H [middle]) 500 nm; (E [right] and H [right]) 200 nm; (F and H [left]) 5 µm; (G) 1 µm. Single blue asterisk (*) represents a sinus space. Double blue asterisks (**) represent a crypt space. Features shown are from a middle-aged adult. Data summarize findings in >30 granulations from frontal pole AG of three decedents and are from more than two independent experiments.

Human AG abut dural tissues (Fig. 1, A and B), yet their dissection at frontal poles resulted in spontaneous dural separation in 14 of 20 specimens (70%). Generally, AG display a basilar stalk with a body that consists of a core, capsule, and one or more apical dome region(s) (Fig. 1 C and Fig. S1; Jayatilaka et al., 1965a; Jayatilaka et al., 1965b). On a screen for histologic and cytologic markers, peripheral AG regions labeled for collagen, arachnoidal, macrophagic, and vascular endothelial markers, but were negative for neuronal, lymphatic endothelial, astrocytic, aquaporin, and other immune cell and/or erythrocyte labels (Fig. 1, D and E). Evaluation of AG cores showed that AG interiors are variably positive for collagen, arachnoidal, macrophagic, mast cell, lymphocytic, plasmacytic, vascular endothelial, and, rarely, synaptic markers (Fig. 1, D and F). As with capsules, cores were negative for astrocytic, neutrophilic, lymphatic endothelial, aquaporin, and/or other neuronal cell markers (Fig. 1, E and F). CD235a erythrocyte label was present within occasional AG cores. Capsule and core labels in an example AG are shown in Fig. 1 D, and label profiles of frontal pole AG from all decedents are summarized in Fig. 1, E and F.

### AG are heterogeneous in morphology

**Figure S2.**
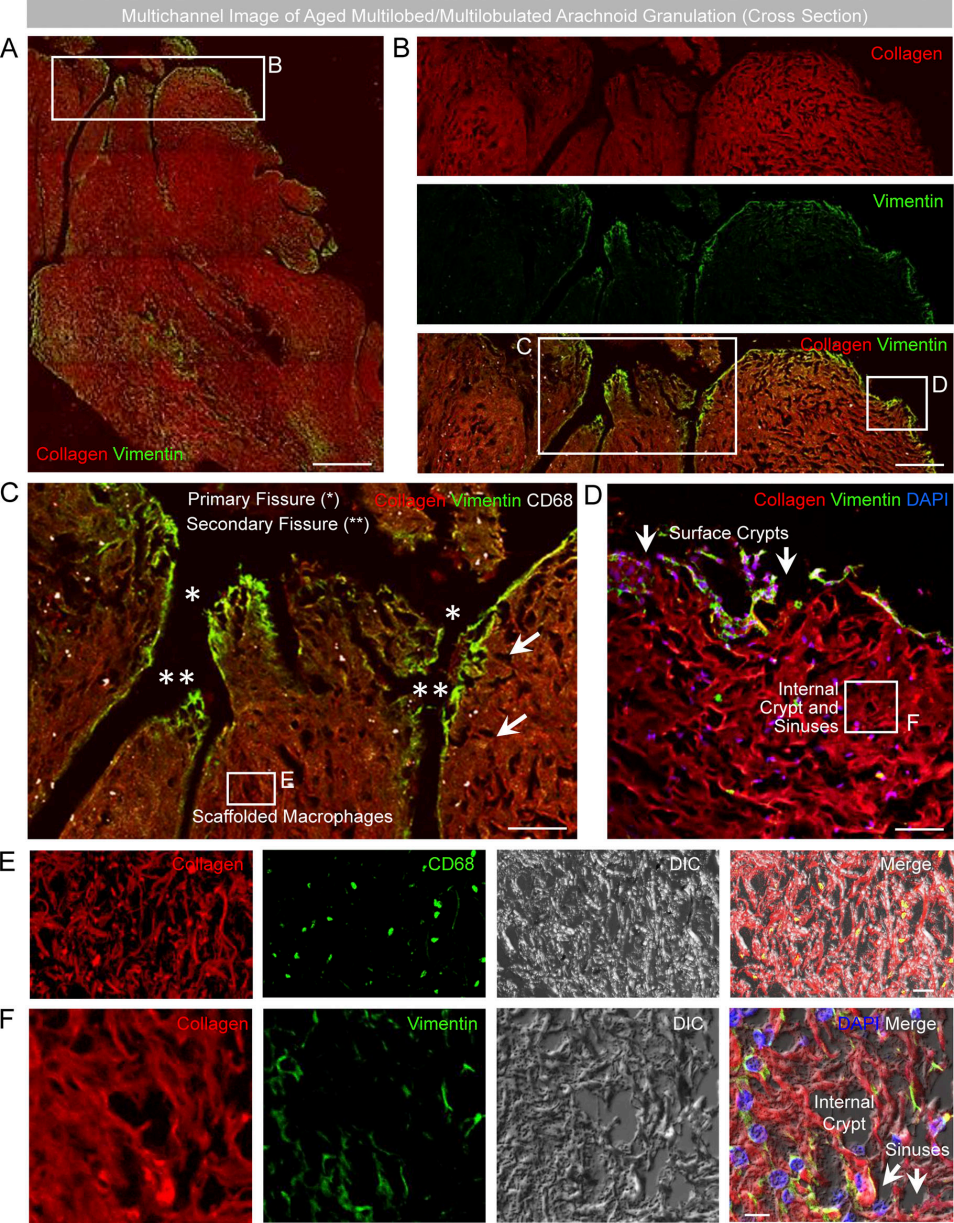
**Fluorescent images of a multilobed AG depict relationships of fissures, sinuses, and crypts within the irregular stromal cavernous space network. (A)** Multichannel images of a multilobed AG, shown in cross section, demonstrate its irregular morphology. **(B)** Cropped images of one edge show multiple indentations at the capsular surface. **(C)** The larger indentations with vimentin-positive lining delineate meningothelium-coated fissures, which impart distinct lobes. **(D)** Smaller surface apertures represent sinuses and crypt openings that are shown in communication with the periapical space. **(E and F)** Internal CD68^+^ macrophages (C and E) and sinuses and crypts (F) are depicted in multichannel images. **(A–F)** Green/FITC, vimentin or CD68; red/CY3, pan-collagen; white/CY5, CD68; blue, DAPI; DIC, differential interference contrast. Scale bars: (A) 100 µm; (B) 50 µm; (C) 25 µm; (D–F) 10 µm. Single asterisks (*) represent primary fissures. Double asterisks (**) represent secondary fissures that branch off of primary fissures. Arrows represent sinus and crypt spaces. Images are confocal z-stacks. Note that internal cavernous spaces are formed by smaller sinuses and larger, lake-like crypts that communicate directly with the periapical region at multiple foci of surface capsular deficiency. Data are representative of multilobed morphology from eight old decedents, studied on >75 independent experiments.

Assessment of frontal pole morphologies showed that AG are polypoid (i.e., pedunculated with narrow-based neck; 289 of 400 AG, i.e., 72%) or sessile (i.e., plaque-like with broad-based neck; 111 AG, i.e., 28%; Fig. 1 G) and heterogeneous in size (Fig. 1 G). Variable numbers of primary surface fissures were present on polypoid AG and gave rise to irregular and asymmetrical lobation (Fig. 1 G). Polypoid AG consisted of unilobed (90 [23%] AG in 17 [85%] participants), bilobed (64 [16%] in 19 [95%]), trilobed (42 [11%] in 16 [80%]), or multilobed (93 [23%] in 16 [80%]) morphologies (Fig. 1 G). Per person, the mean number of unilobed, bilobed, trilobed, multilobed, or sessile AG types, respectively, were 4.5 ± 5.08 (range, 0–14); 3.2 ± 2.26 (range, 0–8); 2.1 ± 1.94 (range, 0–7); 4.65 ± 3.9 (range, 0–10); 5.55 ± 3.78 (range, 0–13). Secondary and tertiary fissures arising from primary fissures were observed on some AG (Fig. 1 G), which gave rise to secondary lobules (98 [25%] in 20 [100%]) and tertiary lobules (40 [10%] in 14 [70%]). AG lobes and secondary and/or tertiary lobules occasionally exhibited focal bridging of apical regions (23 [6%] in 10 [50%]; Fig. 1 G). The distribution of morphologies and dimensions differed by age (Fig. 1, H–J and L). In young, middle-aged, and old persons, mean AG diameters were 1.8, 2.5, and 4.2 mm (overall, 2.9 mm), and median stalk diameters were 138, 215, and 300 µm (overall, 212 µm), respectively. In these same groups, unilobation was seen in 63, 13, and 6% (overall, 22%); secondary lobulation was found in 8, 17, and 51% (overall, 24%); and tertiary lobulation was seen in 1, 5, and 20% (overall, 10%). Multilobed and sessile AG exhibited larger overall and stalk diameters (Fig. 1, K and M). The three-dimensional (3D) and the ultrastructural anatomy of a unilobed, polypoid AG are shown in Video 1 and Fig. S1, respectively, while 3D and cross-section anatomy of multilobed AG with internal fissures are depicted in Video 2 and Fig. S2.

**Video 1.** **Pedunculated AG with bridging.** Vimentin/Pan-Collagen CLARITY image (15×).

**Video 2.** **Sessile AG with fissures.** Vimentin/Pan-Collagen CLARITY image (15×).

### AG capsules are perforated and composed primarily of meningothelium

**Figure 2.**
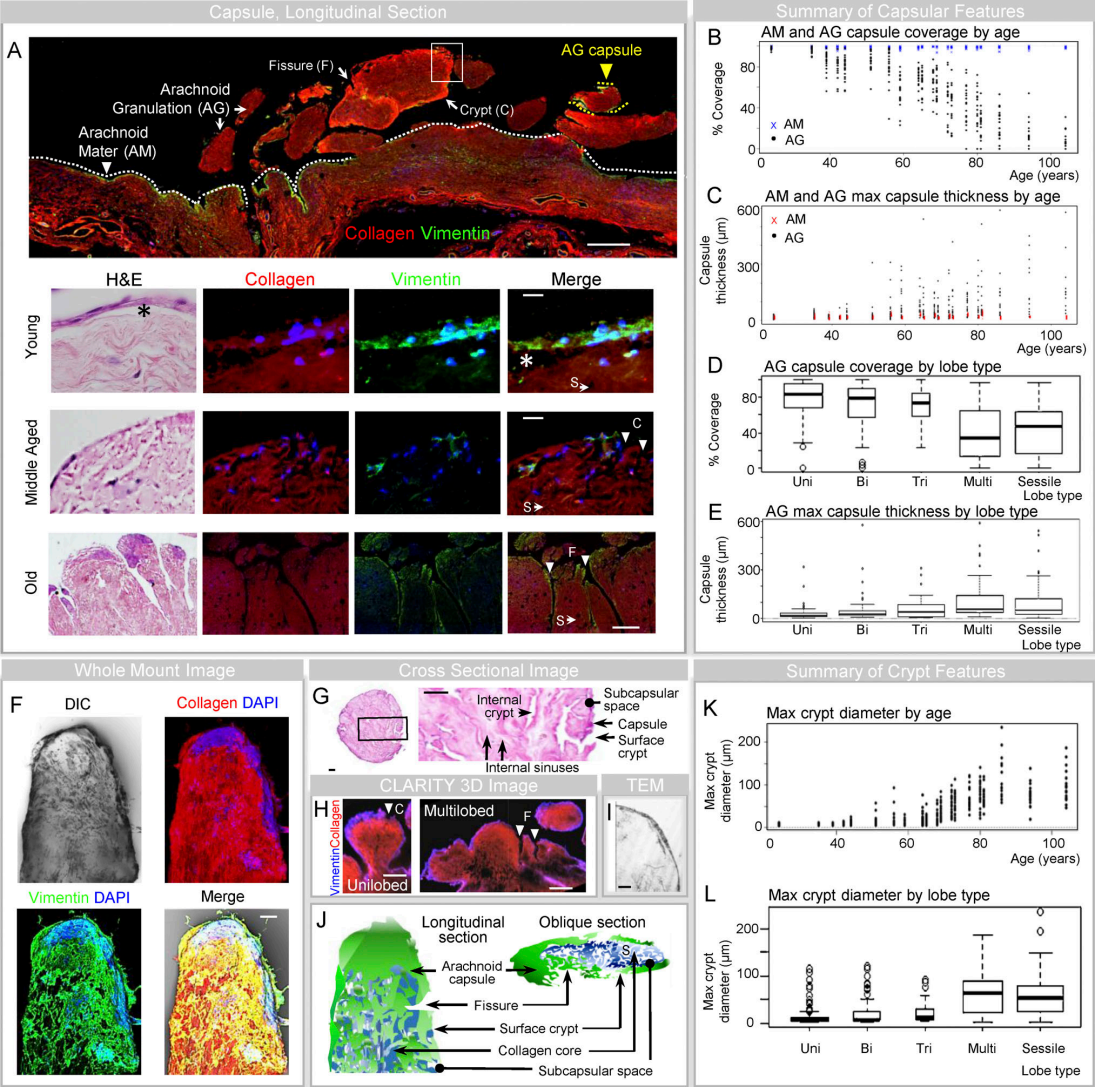
**The meningothelial AG capsule is irregularly perforated by fissures, sinuses, and crypts. (A)** Low-power montage image of human leptomeninges highlights vimentin^+^ meningothelium covering collagen of arachnoid mater (AM, white arrowhead) and AG cores (AG, yellow arrowhead). In the lower panels, AG capsules are shown in young, middle-aged, and old individuals. **(B)** The percentages of vimentin label (i.e., % AM and AG coverage) are shown for each person according to age. **(C)** The capsule thicknesses (i.e., maximum thickness of vimentin^+^ layer) for AM and AG are summarized for each person. **(D)** The percent coverage of AG is additionally summarized according to lobe type. **(E)** AG arachnoid thickness is summarized according to lobe type. **(F)** Multichannel image of an AG lobule is shown and depicts multifocal capsular interruption. **(G)** As shown on an H&E-stained cross-section of a unilobed AG, the capsular interruptions correspond to surface sinus and crypt openings. **(H–J)** Capsules, fissures, and/or crypts are further demonstrated on 3D CLARITY (H), TEM (I), and schematic (J) images. **(K and L)** Maximum crypt diameters are shown according to age (K) and lobe type (L). **(A, F, and H)** Red/CY3, pan-collagen; green/FITC, vimentin; blue, vimentin or DAPI; DIC, differential interference contrast. Scale bars: (A [upper and lower panels]) 500 µm; (A [second panel] and F) 10 µm; (A [third panel] and G) 20 µm; (H) 200 µm; (I) 2 µm. Bi, bilobed; C, crypt; F, fissure; Max, maximum; Multi, multilobed pedunculated; S, sinus; Tri, trilobed; Uni, unilobed. Multichannel images represent confocal Z-stack images. The asterisks (*) in A denote the subcapsular space*.* Vimentin^+^ areas also expressed e-cadherin and epithelial membrane antigen (EMA) in similar pattern and displayed nuclear pseudoinclusions, indicative of arachnoid cells. Additional images from the lower panel of A are shown in Fig. S2. Data represent mean or median values from 400 frontal pole granulations from 20 decedents and are from more than two independent experiments.

Importantly, AG were variably encapsulated as they displayed partial or complete capsules composed of unilaminar, bilaminar, or trilaminar ensheathments (Fig. 2 A). Most AG were ensheathed by a single, irregular covering composed of meningothelium (i.e., arachnoid cells; Fig. 2 A and Fig. S1). The surface lining contained occasional psammoma bodies and frequent intranuclear pseudoinclusions, confirming their cytologic composition. This superficial syncytial capsule rested on loose collagen tissue stroma but was unassociated with basement membrane components (Fig. 2 A and Fig. S1). Dense fibrous connective tissue consistent with dura was rarely associated with the AG margin and, when present, was often only focally adherent to one side. Vascular endothelial ensheathment was observed only on AG that possessed dural covering, and dural covering was only observed on AG that exhibited meningothelial ensheathment. Overall, 371 AG (93%) exhibited encapsulation incorporating meningothelial cells; 354 (89%) exhibited unilaminar encapsulation by meningothelial cells, only; 4 (1%) exhibited evidence of bilaminar or trilaminar encapsulation that was incomplete; and 13 (3%) exhibited trilaminar encapsulation with complete meningothelial, dural, and endothelial coverage. Notably, 29 AG (7%) exhibited no encapsulation by meningothelium, vascular endothelium, and/or dura.

Where present, the meningothelial capsule was finely or coarsely perforated (Fig. 2 A). Features of unilaminar, meningothelial-lined AG are shown in Fig. 2, F–I and depict scattered surface depressions or craters that we refer to as surface crypts (Fig. 2 J; Jayatilaka et al., 1965a; Jayatilaka et al., 1965b). In meningothelial-lined AG, the percent coverage was variable (i.e., 3–100%) and the degree of coverage was associated with age and morphology (Fig. 2, B–E). The median coverage was 90% in young persons, 71% in middle-aged persons, and 27% in old persons (median, 64%). Low coverage (i.e., <67%) was observed in 6% of young persons, 40% of middle-aged persons, and 96% percent of old persons (overall, 54%), while substantial coverage (>90%) was observed in 48% of young persons, 10% of middle-aged persons, and 0% percent of old persons (overall, 16%). Percent meningothelial coverage is summarized according to age in Fig. 2 B, and it was inversely correlated with stalk diameter (−0.94) and maximum crypt size (−0.93). The percent capsular coverage was also associated with lobe type and the number of fissures (Fig. 2, A, D, and E). The maximum surface crypt diameter for AG is summarized according to age and lobe type in Fig. 2, K and L, respectively. Despite marked differences in meningothelial coverage in AG of persons of different ages, meningothelium was intact on arachnoid mater adjacent to AG in all subjects (Fig. 2, B and C).

Though the degree of meningothelial coverage diminished on enlarged AG, residual capsular elements were irregularly thickened and variable with age (Fig. 2 C). The median arachnoid thickness was 18 µm in young persons, 44 µm in middle-aged persons, and 62 µm in old persons (overall median, 40 µm). Thin meningothelial capsules (i.e., width <40 µm) were present in 90% of young persons, 46% of middle-aged persons, and 26% of old persons (overall, 51% of AG), while thick meningothelial capsules (i.e., width >80 µm) were present in 1% of young persons, 20% of middle-aged persons, and 44% of old persons (overall, 23% of AG). Capsular thickness is summarized according to age and lobe type, respectively, in Fig. 2, C and E.

### AG stroma and immune cell composite change with age

**Figure 3.**
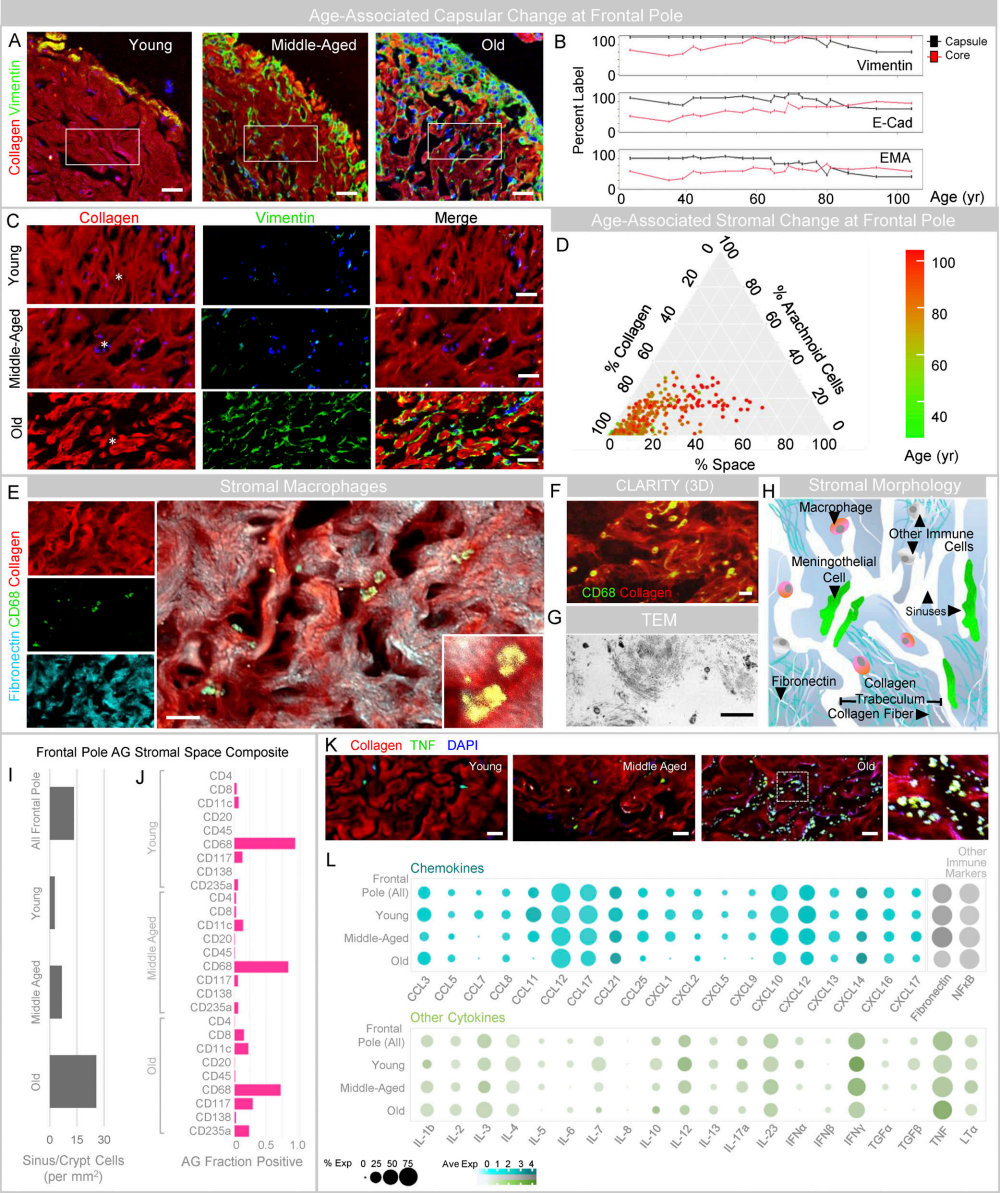
**The framework and composition of AG stroma change with age. (A)** Fluorescent images of AG, shown at intermediate power from young, middle-aged, and old individuals demonstrate collagen-enrichment within stromal cores. **(B)** Ingrowth of capsular elements is more pronounced in older individuals, as demonstrated in a plot depicting percent label of arachnoid markers in capsule and core regions according to age. **(C)** Enlarged cropped images of the core show collagen arrangement into fibrillar and trabecular patterns, which give rise to internal sinus and crypt spaces. **(D)** The AG core composite is summarized based on percent stromal label in an age-coded barycentric plot and illustrates the diminishment of collagen and enhancement of space content with age. **(E–H)** Cavernous space immune cells are shown in confocal (E), 3D CLARITY (F), TEM (G), and schematic (H) images. **(I and J)** Quantification of DAPI label in vimentin^−^ AG core regions is shown in all, young, middle-aged, and old persons (I), and presence of immune cells (i.e., the fraction of AG positive for marker) is shown in J. **(K)** TNF label within sinuses and crypts is further demonstrated in AG from young, middle-aged, and old decedents. **(L)** Semiquantitative cytokine and immune label results are summarized. **(A, C, E, F, and K)** Green/FITC, vimentin, CD68, or TNF; red/CY3, pan-collagen; cyan/CY5, fibronectin; blue, DAPI. Scale bars: (A) 50 μm; (C) 20 μm; (E, F, G, and K) 10 μm. The asterisks (*) in C denote sinus spaces. Data represent findings in 400 frontal pole granulations from 20 decedents and are from more than two independent experiments.

Evaluation of AG cores revealed a stromal framework comprised of connective tissues and irregular cavitary spaces (Fig. 3 A). Within meningothelial-lined AG, a subcapsular space was variably seen between the AG capsule and the core, appearing as a cell-devoid crescentic space in 267 AG (72%; Fig. 2 A, asterisks; Conegero and Chopard, 2003). Within the core, collagen was arranged in fibrillar and trabecular patterns (Fig. 3, A and C). Cavities between collagen fibers, fibrils, and trabeculae formed irregular labyrinthine spaces (Figs. S1 and S2). Serial sections demonstrate communication of smaller interfibrillar sinus spaces with wider intertrabecular tributaries that we refer to as internal crypts (Figs. S1 and S2; Jayatilaka et al., 1965a; Jayatilaka et al., 1965b). The internal crypts branched irregularly and their peripheral tributaries adjoined to surface crypts (Fig. 2 G and Fig. S2). In AG with incomplete meningothelial capsules, the internal spaces (i.e., fissures, sinuses, and crypts) communicated directly with periapical tissue and/or spaces (Fig. S2). In AG with substantial meningothelial capsule, the internal spaces communicated with the subcapsular space that was in a continuum with periapical tissue and periapical space(s) through microscopic capsular fenestrations. In young, middle-aged, and old persons, the maximum crypt diameters (median values) were, respectively 7.5, 16, and 72.5 µm (overall, 23 µm); in these same groups, the percent of individuals with small crypt diameters (i.e., median <10 µm) were 86, 37, and 0% (overall, 34%), and the percentage of individuals with large crypt diameters (i.e., median >30 µm) were 14, 41, and 8% (overall, 44%).

The internal collagen and spaces imparted a sponge-like, cavernous appearance within AG (Fig. 3, A, C, and E; and Fig. S2). Arachnoid cells extended inwardly from areas of capsular deficiencies, enwrapping collagen fibers and trabeculae as well as some fissures, sinuses, and crypts (Fig. 3 A and Figs. S1 and S2). Prominent changes in stromal collagen, arachnoidal, and space composite were seen with age (Fig. 3, A–C). These components, respectively, constituted 93, 2, and 6% of core area in young persons; 91, 3, and 6% in middle-aged persons; and 67, 14, and 19% in old persons (overall mean, 79, 8, 13%). A barycentric plot summarizes the composition of the stromal framework in 400 frontal pole AG according to age (Fig. 3 D). Small, endothelial-lined, blood-containing vascular structures, i.e., capillaries (0.75%/10%) and nerve fibers (0.5%/10%) were histologically confirmed within the connective tissue core but were not found in capsules or subcapsular spaces.

Within cavernous spaces, degenerated cells and cell elements were present and were admixed with blood, mononuclear immune cells, and chemokine and cytokine labels (Fig. 3, E–H). Cavernous space content in young, middle-aged, and old decedents is summarized in Fig. 3, I–L. Degenerated and immune cell content increased with age (Fig. 3, I–K). A majority of cavernous space cells were histologically confirmed as CD68-positive tissue macrophages (Fig. 3, E–H; and Video 3). Other cavernous space cells included CD117^+^ mast cells; CD11c^+^ dendritic cells; CD4^+^ and CD8^+^ T lymphocytes; CD20^+^ B lymphocytes; CD138^+^ plasma cells; and CD256a^+^ erythrocytes (Fig. 3 J and Fig. S2). Lymphocyte and plasma cell densities were variable, i.e., 17–46 lymphocytes/mm^2^ and 2–13 plasma cells/mm^2^, but were present in only few AG (1%/15% and 1%/10%, respectively; Fig. 3, G–L; and Fig. S2). Macrophages ranged in number from 1 to 170 per AG. The median macrophage density was 5 cells/mm^2^ in young persons, 1 cell/mm^2^ in middle-aged persons, and 1 cell/mm^2^ in old persons (overall, 1 cell/mm^2^). The percentages of AG with dense macrophages (i.e., >2 cells/mm^2^) were 73, 24, and 1% for these same groups (overall, 27%), while the percentages with sparse macrophages (i.e., <0.5 cell/mm^2^) were 6, 41, and 82%, respectively (overall, 49%). The macrophage cell density correlated directly with percent capsular coverage (r = 0.74), while it was inversely correlated with AG diameter (r = −0.61), crypt diameter (−0.66), and stalk diameter (r = −0.72). Persons with higher mean macrophage cell density tended to have AG with fewer secondary (r = −0.65) and tertiary lobules (r = −0.62). The degree of chemokine, cytokine, and other molecular immune labels also ranged across age groups (Fig. 3, I–L). The degenerated cells and cell elements present within AG crypts and sinuses were prominently labeled with TNF (Fig. 3 K). Across age, CCL3, CCL12, CCL17, CXCL10, CXCL12, fibronectin, NFκB, and IFNγ were also consistently expressed in the stroma, while a range of other cytokines was present at lower levels (Fig. 3 L). Fibronectin and TNF label were increased in AG of middle-aged and old persons, respectively (Fig. 3 L).

**Video 3.** **AG stroma with sequestered macrophages.** Pan-Collagen/CD68, CLARITY image (15×).

### AG are heterogeneous in location and give rise to distinct types based on dome position.

**Figure 4.**
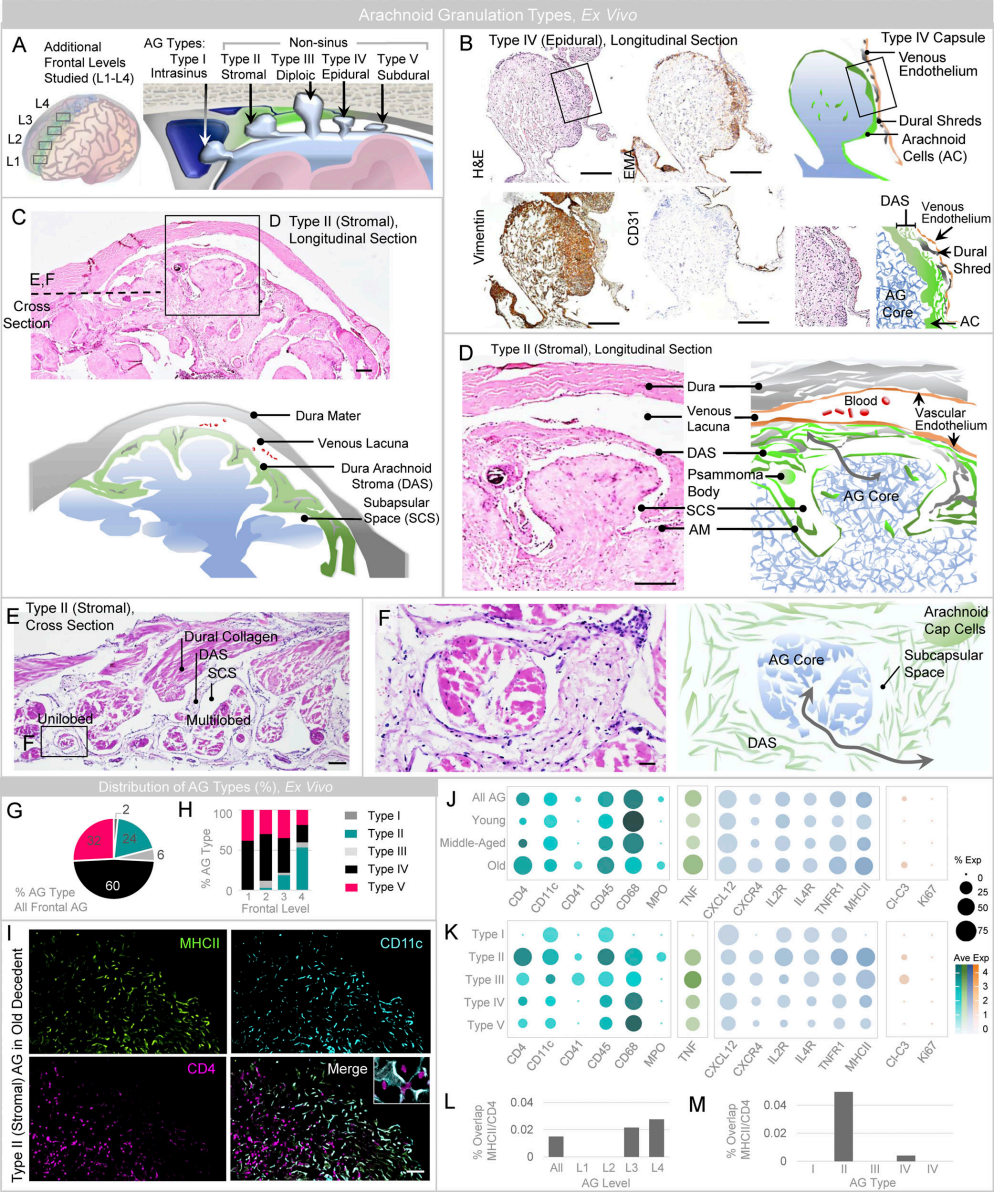
**Sinus and nonsinus AG types are observed on postmortem examination. (A)** A schematic depicts frontal levels studied on expanded frontal convexity sampling (left), and variable AG dome positions identified (right). **(B)** Intermediate-power image of the frontal perisinus region demonstrates epidural type (i.e., Type IV) AG, in this case with partial trilaminar ensheathment. H&E-stained section shows well-defined meningothelial AG capsule with partial ensheathment by dural connective tissue and DVS enthothelium, as confirmed by immunoperoxidase labels for vimentin, epithelial membrane antigen (EMA), and CD31. A cropped image of the granulation edge, shown on the lower right-hand side, depicts the trilaminar capsular investments. **(C)** Another image demonstrates stromal type (i.e., Type II) AG, with dome embedment within DAS. **(D–F)** Enlargements of cropped AG images in longitudinal (D) and cross section (E and F) orientation are shown. The enlarged images depict DAS envelopment of the apical AG dome region and communication of the AG core with the subcapsular space and DAS (gray arrows). **(G)** AG types present along frontal convexities are summarized. **(H)** AG types present at distinct frontal levels are summarized. **(I)** MHCII-expressing APC and CD4-expressing T cells are depicted in sinuses and crypts of a Type II AG, with multifocal label overlap suggesting the presence of immune synapses (inset). **(J and K)** Results of select immune cell, cytokine, chemokine and other labels are summarized according to age (J) and AG type (K). **(L and M)** The percent overlap of MHCII (APC) and CD4 (T cell) label, suggestive of immune synapses, is summarized at frontal levels (L) and in distinct AG types (M). **(I)** Green/FITC, MHCII; violet/CY3, CD4; cyan/CY5, CD11c. Scale bars: (B–D) 200 µm; (E) 100 µm; (F and I) 50 µm. Histological features depicted are from a middle-aged adult. Data summarize findings in 124 granulations from frontal convexities of 15 decedents and are from more than two independent experiments.

**Figure 5.**
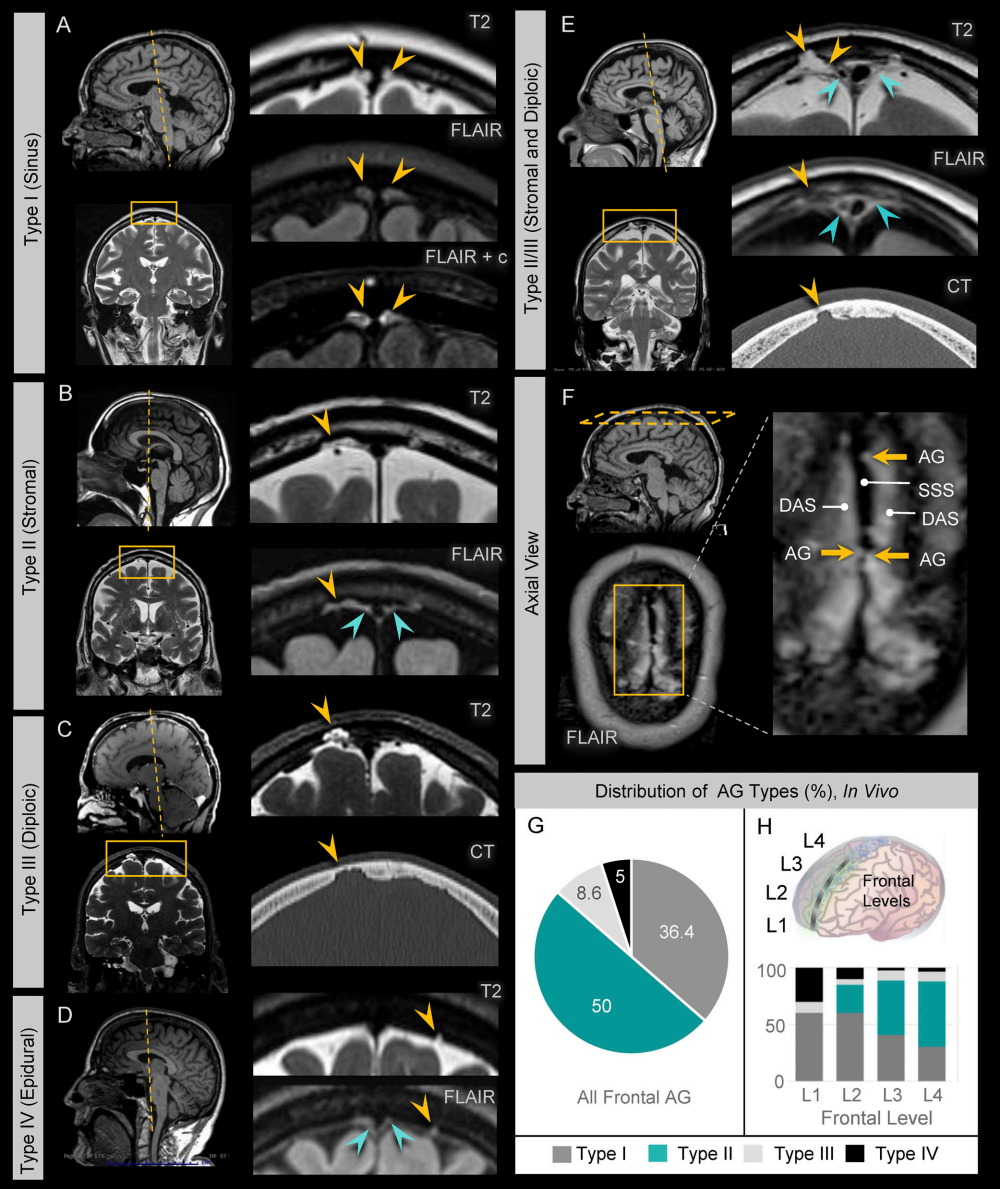
**Sinus and nonsinus AG types are confirmed by in vivo MRI.** MRI examination in young, middle-aged, and old persons confirm the presence of sinus and nonsinus granulations. **(A)** Type I AG have domes (yellow arrows) located at midline abutting the DVS. **(B)** Type II AG have domes (yellow arrows) located within dural stroma, which is visualized on FLAIR images as hyperintense perisinus soft tissue (green arrows). **(C)** Type III AG have domes (yellow arrows) located within skull diploe and are associated with cortical defects of the inner calvarium. **(D)** Type IV AG have domes (yellow arrows) that are neither intrasinus nor intrastromal in location and abut dura without eroding the calvarial cortex; Type V AG are not definitively observed in this series. **(E)** Multilobulated hybrid AG types were also occasionally observed, as shown in this example of a Type II/III AG that displayed both stromal and diploic dome embedment. **(F)** Axial FLAIR image at the calvarial vertex shows superior sagittal sinus (SSS) in the midline with surrounding FLAIR hyperintensity due to a combination of proteinaceous DAS and Type I and Type II AG. Note irregularity of the SSS at the site of Type I AG. **(G and H)** The distribution of AG types is summarized along the frontal convexities (G) and (H) at distinct frontal levels and highlight a greater percentage of stromal type AG at posterior regions. Data represent 220 frontal granulations from 15 subjects.

We next asked whether there are differences in AG position along the frontal convexities. To assess this, AG were analyzed at four levels along frontal lobes, i.e., numbered L1–L4 from anterior (frontal pole) to posterior (frontal vertex), respectively (Fig. 4 A, left). Evaluation revealed distinct AG types based on the location of AG dome embedment (Fig. 4 A, right). Type I consisted of intrasinus AG that have been previously described (Kida et al., 1988; Weller et al., 2018). Four nonsinus AG types were also seen: Type II demonstrates dome embedment within the dural stroma (i.e., stromal; Fig. 4, A and C); Type III demonstrates dome protrusion completely through the dura, resulting in a dural defect (i.e., transdural); Type IV demonstrates dome abutment along the dura, without dural defect (i.e., epidural; Fig. 4, A and B); and Type V demonstrates AG domes that are unassociated with dura (i.e., subdural). Capsules of Type II AG were notably distinct from capsules of other AG types in that their meningothelial linings were admixed with loose dural connective tissue, forming dura-arachnoid stromal capsules that had distinct qualities (Fig. 4, D and E) described below. To correlate ex vivo and in vivo findings, AG anatomy was also examined using in vivo magnetic resonance imaging (MRI). Diverse AG types were identified along frontal convexities of healthy individuals who were neurologically intact (Fig. 5). These included Type I, which demonstrates abutment of the superior sagittal sinus by the AG dome (Fig. 5 A); Type II, which demonstrated AG dome embedment within the dural stroma (Fig. 5 B); Type III, which demonstrates protrusion of AG domes through dura mater and inner calvarial cortex with insertion into diploic spaces (Fig. 5 C); and Type IV, that exhibited AG dome abutment into dura mater without evidence of dural or calvarial cortical defects (Fig. 5 D). While Type V (subdural) AG were not definitively identified in live persons, hybrid types were observed by MRI and involved multilobated AG (Fig. 5 E). Thus, MRI inspection confirmed that in vivo locations of AG were not confined to the DVS and verified the heterogeneity of nonsinus AG locations. Notably, ex vivo histology and in vivo MRI analyses both revealed a predominance of Type II AG along posterior frontal convexities (Fig. 4, G and H; and Fig. 5, F–H). In this series, 50% of all AG examined in frontal convexities were classified as Type II (i.e., stromal type) in vivo, whereas only 19% were classified as this type by histology. This discordance may be associated with heterogeneous biological factors, other variables associated with AG sampling or harvesting, and/or potential postmortem AG deflation.

### A subset of AG is enriched with antigen-presenting and bone marrow–derived cells

In light of the above findings, we next studied select immune, receptor, cell death, and proliferation markers in various AG types along the frontal convexity. It was discovered that DAPI nuclear label was increased in vimentin^−^ regions of Type II and Type III AG, relative to similar regions of other AG types. Across frontal convexities, cavernous space cells were also increased in AG of old persons (Fig. 3, C and I; and Fig. 4, I and J). There was enrichment of CD4^+^ and CD45^+^ lymphocytes and MHCII^+^ APC in Type II AG and AG of old persons (Fig. 4, J and K). Interestingly, myeloperoxidase^+^ neutrophils were observed in some Type II AG of old persons at posterior frontal levels. Cleaved-caspase 3^+^ apoptotic bodies were present in some Type II and Type III AG of old persons, although Ki67, a proliferation marker, was negative in all AG types of all persons. TNF predominantly labeled degenerated cells and cell elements within AG crypts and sinuses and was enriched in nonsinus AG of old persons, whereas TNFR1 label was most notable in Type II AG and AG of old persons. Despite an increase in TNF, TNFR1, and MHCII labels in select AG types, CD68 macrophage label was not increased in the corresponding AG. Within some AG, the expression of the dendritic cell (CD11c) and APC (MHCII) label was observed in close proximity to CD4 T cell label within crypts and sinuses (Fig. 4 I). In rare AG, there was the adherence of CD4^+^ T cells with CD11c^+^/MHCII^+^ APC, consistent with immune synapses (Fig. 4 I, inset). The mean percent of CD4 and MHCII label overlap was 0.0059 in young persons, 0.011 in middle-aged persons, and 0.016 in old persons (overall, 0.012%), being higher in posterior frontal and Type II AG (Fig. 4, L and M). On a screen, CD41^+^ megakaryocytes were also discovered in rare Type II and Type III AG of old persons (Fig. 4, J and K; Sun et al., 2021), suggesting that bone marrow–derived elements have the potential to permeate AG (Mazzitelli et al., 2022). Overall, the densities of TNF, MHCII, CD4, CD68, CD41, and cleaved-caspase 3 labels were greater in nonsinus versus intrasinus AG, though AG of all types showed expression of CXCL12 across age (Fig. 4, J and K).

### Perisinus stroma is enriched with diverse immune components

**Figure S3.**
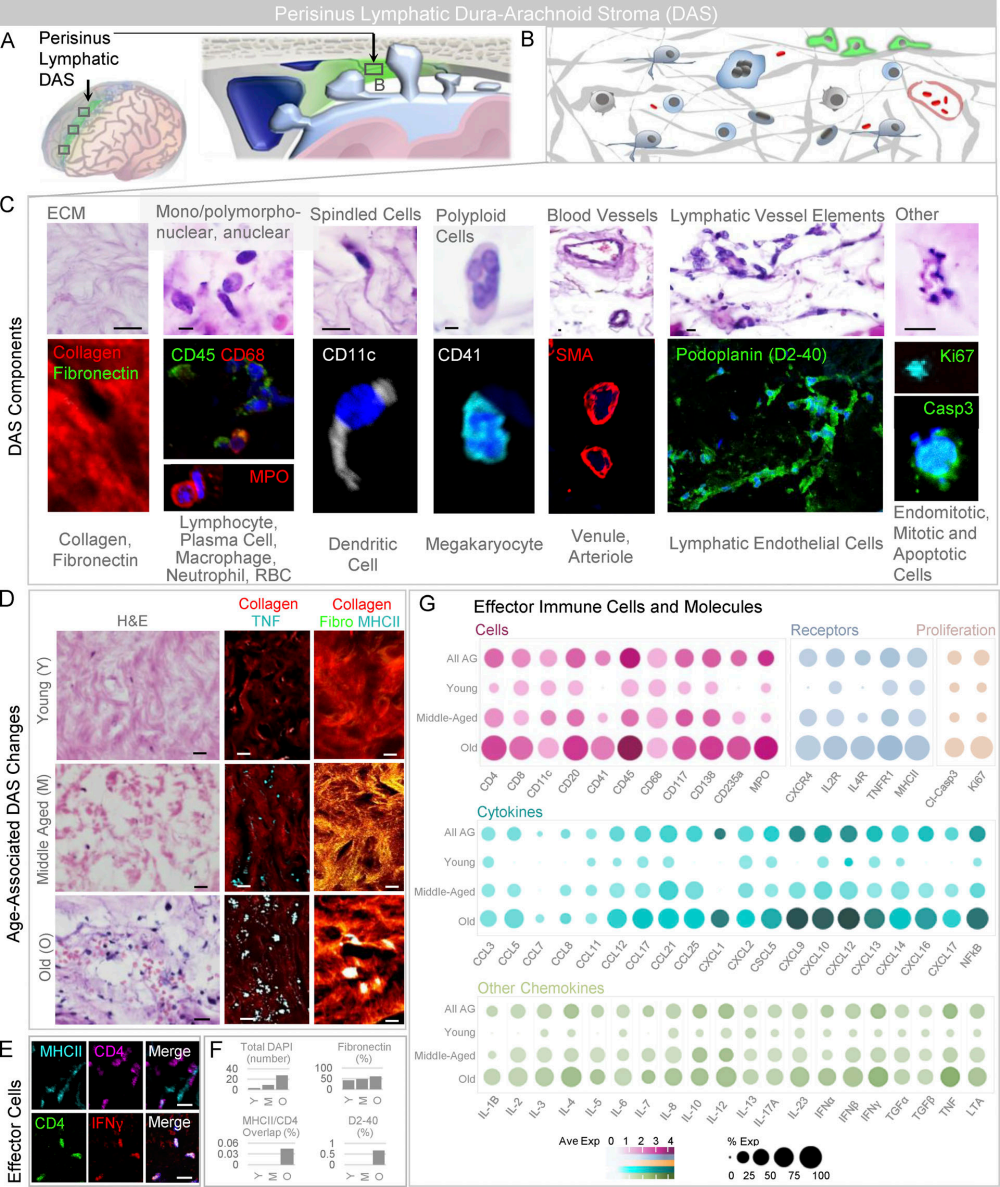
**The perisinus DAS harbors mixed immune cells, immune synapses, and lymphatic endothelial components. (A)** The perisinus region houses DAS. **(B)** This stroma contains extracellular matrix elements, vessels, and heterogeneous cell types. **(C)** Cropped intermediate-power H&E-labeled and immunofluorescent images of the stromal tissue reveals collagen and fibronectin-rich matrix admixed with immune cells, proliferating and degenerating cells, and blood vascular and lymphatic vascular elements. **(D)** Intermediate-power images of DAS from young, middle-aged, and old individuals on routine stain (left) and with pan-collagen and TNF label (middle) or pan-collagen, fibronectin, and MHCII labels (right). **(E)** In addition to trapped TNF-positive cells and cell debris, effector immune cells are observed within DAS of an old decedent. **(F)** DAS cellularity, percent fibronectin label, percent overlap of MHCII and CD4 labels, and percent D2-40 label are summarized in young (Y), middle-aged (M), and old (O) decedents. **(G)** Additional cellular, cytokine, chemokine, and molecular label results are summarized according to age, and highlight heterogeneity of immune components in DAS tissue. **(C–E)** Green or violet/FITC, fibronectin (fibro), CD45, podoplanin, cleaved-caspase 3, Ki67, or CD4; red/CY3, pan-collagen, CD68, myeloperoxidase, SMA, or IFN-γ (γ); white or cyan/CY5, CD11c, CD41, TNF, or MHCII; blue, DAPI. Scale bars: (C and D [right]) 5 µm; (D [left and middle] and E) 10 µm. Data summarize findings in 27 DAS sections from nine decedents and are from >two independent experiments.

Given the enrichment of immune cells in Type II AG that traverse dura-arachnoid stroma (DAS), we next studied this perisinus tissue (Fig. S3 A). We discovered that DAS consists of irregular, loose collagen and fibronectin-rich tissue with a variable number of arachnoid cells and also harbors diverse immune cell populations (Fig. S3, B and C; Rustenhoven et al., 2021). A systematic screen revealed the presence of CD11c^+^ dendritic cells; CD4 and CD8^+^ T cells; CD20^+^ B cells; CD138^+^ plasma cells; CD68^+^ macrophages; CD117^+^ mast cells; and myeloperoxidase^+^ neutrophils within this region (Fig. S3 C). Interestingly, CD235a^+^ erythrocytes and CD41^+^ megakaryocytes were also prominent in DAS, indicating extravasation of blood cells and bone marrow–derived elements into the region (Mazzitelli et al., 2022). In some specimens, diverse cytokine and chemokine labels (Fig. S3 G) and scattered nonlumenized D2-40 (podoplanin) expressing lymphatic endothelial cells (Fig. S3 C) were also haphazardly arranged herein along with cleaved-caspase 3^+^ apoptotic bodies, Ki67^+^ endomitotic and mitotic bodies (Fig. S3 G), and degenerated TNF^+^ cells and cell elements (Fig. S3, D, F, and G). DAS cellularity was increased in old decedents (Fig. S3, D–G). Notably, CD4, MHCII, and IFNγ labels all increased with age, as did the overlap of CD4 and MHCII labels (Fig. S3, D–G), suggesting that DAS is a site of innate and adaptive immune signaling (Rustenhoven et al., 2021). On MRI, this perisinus stromal tissue is characterized by hyperintense signal on the precontrast FLAIR sequence (Fig. 5, B and E), similar to the cores of Type II AG and consistent with the presence of proteinaceous and cellular exudate material in these regions (Ikushima et al., 1999; Albayram et al., 2022).

### AG in the context of brain fluid dynamics and immunology

In light of AG locations as well as their deficiency of apical basement membrane and/or complete membranous capsules, results of this study suggest that AG are conduits that adjoin the subarachnoid space, subdural space, DVS, DAS, and diploic spaces. Moreover, the AG microstructure, which consists of collagen and fibronectin-rich tissue, highlights an internal adhesive substratum that entraps antigen and facilitates scaffolding of immune cells, likely facilitating their migration, signaling, and associated immune activities (Fernandes et al., 2020). Therefore, outpouched AG structures appear to represent sites for the convergence and sequestration of CNS antigen, efflux fluid, and immune cells. Prior tracer studies have demonstrated fluid transfer phenomena at adjacent perivenous and perisinus junctions in live humans (Eide and Ringstad, 2021; Mehta et al., 2021). Specifically, an MRI study depicts the movement of molecular gadobutrol tracer (∼600 kD) from the CSF to the parasagittal dura focally at the brain vertex, at a region where we observe AG embedment within immune cell–rich DAS tissue. We further confirm the presence of lymphatic endothelial elements in the DAS region (Absinta et al., 2017; Louveau et al., 2015). Collectively, the morphology of AG implies biological roles in antigen filtration and cell trapping, while the presence of cellular stromal infiltrates indicate clearly that these structures are sites for immune activity. Thus, this study supports the hypothesis that perisinus tissues, including AG, are specialized for the processing of CSF egress and form a critical interface for immune surveillance at the brain surface (Rustenhoven et al., 2021).

### AG in the context of aging

In this study, complex morphologic changes were observed in AG of old persons (Fig. 2, F–H). Age correlated strongly with whole AG diameter (0.87; Fig. 2 H), AG stalk diameter (0.92; Fig. 2 J), and the number of AG lobes. For unilobed, bilobed, trilobed, multilobed, and sessile morphologies, the respective correlation with age was −0.85, −0.70, −0.30, 0.86, and 0.83. In combination with the widening of AG stalks, which manifested as increased sessile structures, bridging of AG apices was evident (Fig. 1 I). Thus, AG appeared to merge, dilate, and/or conglomerate in the aged (Fig. 1, H and I). With age, some capsules also denuded to yield AG cores with complete loss of meningothelial coverage (18% of AG from 75% of old persons), whereas AG denudation was not observed in AG of any young or middle-aged persons. Overall, the percent capsular coverage correlated inversely with age (r = −0.95), while the percent capsular label in AG core regions directly correlated with age (0.86; Fig. 3 B). Stromal collagen content also prominently diminished, while stromal space and associated meningothelial cell content increased (Fig. 3, C and D; and Fig. S2). Thus, the AG internal milieu was markedly altered in older persons, possibly related in part to fluid stasis. Despite an overall increase in AG sinus and crypt cell density, macrophage diminishment was also observed within AG of old persons (Fig. 3, I and J). The overlap of MHCII^+^ APC and CD4^+^ T cells was also greater in AG cores as well as DAS of old persons (Fig. 4, I–M; and Fig. S3, E and F). Though the reasons for this are unclear, increased immune cell density and adaptive immune signaling may be associated with immune senescence, fluid stasis, other flow aberrations, and/or overabundance of CSF antigens, among other factors.

### Conclusion

In light of recent clinical and neuroscientific evidence (Ringstad and Eide 2020; Eide and Ringstad, 2021; Rustenhoven et al., 2021), the anatomical and immunohistochemistry data presented here indicate that AG convey cells, cellular debris, fluids, and biomolecules including molecular immune signals and provide regionalized CNS enclosures for innate and adaptive immune signaling, thereby serving as lymph node–like sentinels at brain–meningeal–vascular interfaces (Louie and Liao. 2019). As such, AG structures likely have broad implications in neurophysiology and should be further studied with respect to solute and CSF transport, antigen and protein metabolism, sleep, and general immunity. Results of this study also characterize heterogeneous senescent features of human AG and suggest the need for further investigation of these structures in aging. Collectively, these new histological data provide a revised model for understanding and investigating mechanisms of functional crosstalk between the glymphatic and lymphatic systems in humans. These findings also raise several new questions and hypotheses regarding AG activity in aging, as heterogeneous maturational and degenerative changes may influence the evolution of various neoplastic (Hu et al., 2020), inflammatory (Amor et al., 2014), infectious, traumatic (Needham et al., 2019), developmental, vascular (Mestre et al., 2017), senile and/or neurodegenerative processes and diseases (Da Mesquita et al., 2018b; Mehta and Schneider, 2021). AG and DAS may also represent unique targets for neurodiagnostics and disease monitoring, and potentially, for neuroimmune modulation and/or administration of novel brain therapies.

## Materials and methods

### Human tissues

AG from 20 hospital decedents (mean age at death in years 64.15, SD = 20.64, range 24 to 104) were collected from a tertiary care facility over a period of 5 yr. Specimens were collected from the right superior frontal gyri based on exclusion of intracranial pathologies. Patients died acutely from myocardial infarction, pulmonary embolism, trauma, or arrhythmia. For persons >50 yr in age, β-amyloid and tau were analyzed on the right hippocampus and frontal and parietal cortices to rule out Alzheimers’ disease. Specimens were negative for microscopic and macroscopic brain hemorrhages, acute and chronic microscopic and macroscopic infarcts, anoxic change(s), and contusion(s). At the time of brain removal, AG blocks were harvested via coronal excision. Two tissue sections were dissected en bloc at frontal poles prior to dural removal to preserve relationships of AG with meningeal and vascular elements, including superior sagittal sinus tissues. AG were similarly harvested along the posterior frontal regions of 15 decedents. Specimens were processed per routine protocol including fixation in 10% paraformaldehyde, embedment in paraffin, and serial sectioning at 7 μm thickness. Due to specimen sampling, sectioning yielded AG predominantly in coronal (longitudinal) orientation, with fewer in oblique and axial cross-sections. Slides from each block were stained with H&E and Masson trichrome special stain, and serial sections were collected for immunohistochemistry (*n* = 20). Since frontal pole specimens were serially sectioned to yield 20 AG per decedent, a total of 400 AG were analyzed quantitatively in this region. A single cross-section was studied in posterior frontal blocks, which yielded variable numbers of AG from sampled decedents. Supplemental frontal pole AG blocks were also harvested for analysis by whole mount imaging (*n* = 8), transmission electron microscopy (TEM; *n* = 3), tissue clearing technique (*n* = 5), and immuno-electron microscopy (*n* = 3).

### Immunohistochemistry

Deparaffinized tissue sections were rinsed in graded ethanol and washed with PBS (10 mmol/liter, pH 7.4). For antigen retrieval, slides were placed in citrate buffer (10 mmol/liter, pH 8.0), heated in a microwave oven at 900 W for 10 min, and then washed in PBS. Slides were incubated with a mixture of 5% goat serum (Sigma-Aldrich) and 0.2% Triton X-100 for 1 h at room temperature and then overnight with primary antibodies at 4°C. The next day, the slides were rinsed again in PBS. Various cytological and tissue markers were applied. Primary antibodies used are shown in Table S1. For single labeling, an immunoperoxidase label was developed using biotin-conjugated secondary antibodies. Sections were incubated for 30 min in PBS with 20% Tween containing 0.3% H_2_O_2_ to block endogenous peroxidase activity. Following overnight incubation with primary antibodies, sections were incubated for 2 h with a biotinylated species-appropriate secondary antibody (1:500; Vector Laboratories). After PBS washes, sections were incubated in avidin–biotin solution (Vector Laboratories), and the color was developed in diaminobenzidine (DAB) chromogen solution (0.02% DAB in 0.175 mol/liter sodium acetate) activated with 0.01% H_2_O_2_, using cresyl violet as a counterstain to visualize cell nuclei. The sections were rinsed, mounted, dehydrated, coverslipped with distyrene plasticizer and xylene mounting medium (Electron Microscopy Services), and then imaged. Brain and lymph node tissues were used as positive controls while the omission of primary antibodies and isotype controls were used as negative controls.

To further analyze anatomic relationships, immunofluorescence labeling was performed for positive markers. Slides were incubated with primary antibodies, rinsed in PBS, then incubated at room temperature for 1 h with fluorescently conjugated species–appropriate secondary antibodies (1:500; Alexa Fluor 488, Alexa Fluor 555, or Alexa Fluor 647; Invitrogen/Molecular Probes). Brain and lymph node tissues were used as positive controls while the omission of primary antibodies and isotype controls were used as negative controls. The sections were coverslipped with a polar mounting medium containing antifade reagent and DAPI (Invitrogen), and then imaged.

### Quantification of histologic AG features

Sections were imaged using an epifluorescent (Nikon Eclipse Ni-Ui; Nikon Instruments Inc.) and/or confocal (Olympus FV3000; Olympus America, Inc.) microscope, and AG were visualized as herniations of arachnoid tissue at frontal gyral apices. For quantification of the label, batch images were acquired at 200× or 400× and were systematically processed and analyzed using Nikon Microscope Solutions Imaging Software (NIS-Elements AR Version 4.30.01) or ImageJ (U.S. National Institutes of Health; https://imagej.nih.gov/ij/). Immunofluorescent data were recorded on sections of AG in a longitudinal orientation. The AG were digitally traced and the AG core and edge, incorporating a 10 µm rim, were selected as separate regions of interest. For each AG, whole diameter, stalk diameter, and maximum space diameter were recorded. Given the sensitivity of vimentin for edge labeling, vimentin-labeled sections were used for the analysis of percent capsular coverage and capsule thickness measurements, while pixel and/or object count tool was used to explore cell count or label within individual AG. Cross-section areas of AG were determined and immune cell counts were normalized to AG areas to determine select immune cell densities. Within AG cores, the percent vimentin^+^ and collagen^+^ pixels were analyzed, and unlabeled pixels were determined for extrapolation of space areas. For each marker, the percent pixel label was also determined within different regions of interest and/or brain regions. Analyses were conducted by investigators who were blinded to demographical information and anatomical information and were confirmed by an experienced, board-certified anatomically trained neuropathologist.

### Whole mount imaging

For screening of 3D structure, multiple AG from eight decedents were analyzed by whole mount imaging (young [*n* = 2]; middle-aged [*n* = 3]; and old [*n* = 3]; Olympus FV3000; Olympus America, Inc.). The samples were first blocked for 6 h at room temperature with a mixture of 5% goat serum (Sigma-Aldrich) and 0.2% Triton X-100. They were then passively immunolabeled by incubation for 2 d at room temperature in PBS solution containing 1 mg/ml DAPI (Thermo Fisher Scientific) and rabbit anti-pan-collagen (PA1-85324; Invitrogen) and chicken anti-vimentin (PA1-10003; Invitrogen) primary antibodies. Next, the samples were rinsed in PBS and then incubated at room temperature for 6 h with fluorescently conjugated species-appropriate secondary antibodies (1:500, Alexa Fluor 488 and Alexa Fluor 555; Invitrogen/Molecular Probes). After further rinsing, the tissues were imaged using a confocal microscope (Olympus FV3000; Olympus America, Inc.) to generate Z-stack images.

### Tissue clearing, staining, and imaging

Multiple AG from five decedents (young [*n* = 1], middle-aged [*n* = 2], and old [*n* = 2]) were processed following the application of the SHIELD protocol (LifeCanvas Technologies; Park et al., 2018). The samples were first cleared for several days with SmartClear II Pro (LifeCanvas Technologies), a device based on stochastic electrotransport (Kim et al., 2015), and were then actively immunolabeled within 24 h using SmartLabel (LifeCanvas Technologies), a device based on eFLASH technology that integrates stochastic electrotransport (Kim et al., 2015) and SWITCH (Murray et al., 2015). For labeling, the following primary antibodies were used: chicken anti-vimentin (PA1-10003; Invitrogen), rabbit anti-pan-collagen (PA1-85324; Invitrogen), and/or mouse anti-CD68 (14-0688-82; Invitrogen). Species-appropriate fluorescently conjugated secondary antibodies were applied in 1:2 primary:secondary molar ratios (Jackson ImmunoResearch). The samples were next incubated in EasyIndex (LifeCanvas Technologies) for refractive index matching and then screened using a confocal microscope (Olympus FV3000; Olympus America, Inc.). Images from representative samples were imaged at 15× with a SmartSPIM light sheet microscope (LifeCanvas Technologies) and rendered using Oxford Instruments Imaris 3D (version 9.5).

### TEM

Multiple AG from three middle-aged decedents were fixed in 2.5% glutaraldehyde at 4°C and postfixed in 1.0%/1.5% osmium tetroxide and potassium ferrocyanide for 1 h. The specimens were then rinsed in distilled water, dehydrated in graded alcohol, and transitioned into propylene oxide/resin followed by 100% resin. Polymerization was carried out at 60°C for 24 h. AG were isolated from the embedded sample and mounted onto epoxy blocks and then sectioned at 1 mm thickness using an ultramicrotome and mounted on glass slides then stained with 1% toluidine blue. Following verification of AG structures by light microscopy, an ultramicrotome and a diamond knife were used to cut 70-nm ultrathin sections for placement onto formvar/carbon-coated nickel slot grids. The grids were stained with aqueous uranyl acetate and lead citrate and then imaged using a Hitachi 7650 transmission electron microscope and Gatan 11 megapixel Erlangshen digital camera for digital 649 capture using Digitalmicrograph software.

### Immuno-electron microscopy

Immuno-electron microscopy labeling was performed on multiple AG from three middle-aged decedents using a pre-embedding technique. The samples were permeabilized in 0.1% Triton X-100 with 5% goat serum (Sigma-Aldrich) for 2 h and then incubated for 2 d at 4°C with a monoclonal mouse-anti-CD68 antibody (14-0688-82, 1:50; Invitrogen, Thermo Fisher Scientific). Following PBS rinse, samples were incubated overnight at 4°C in preadsorbed biotin goat anti-mouse secondary antibody (ab97033, 1:200, Abcam). They were then rinsed and incubated in the dark in ExtrAvidin Peroxidase (2886, 1:100; Sigma-Aldrich) for 90 min, rinsed again in PBS and TRIS buffer, presoaked in 0.6% DAB/TRIS (25 min), and treated with DAB/hydrogen peroxide (0.03%) solution (8 min). Following additional rinses in TRIS and 0.1 M sodium cacodylate buffer, sections were fixed overnight at 4°C in 2.5% glutaraldehyde in 0.1 M sodium cacodylate buffer. After further rinsing in 0.1 M sodium cacodylate buffer and double distilled water, the sections were incubated at 60°C in a 0.2% silver nitrate solution (10 min) to intensify the DAB label. They were next rinsed in ddH_2_O followed by 0.05% gold chloride, ddH_2_O, aqueous 2.5% sodium thiosulfate, and ddH_2_O before being postfixed in 1.0% osmium tetroxide and 0.1 M sodium cacodylate buffer (30 min). After a final rinse in buffer and then ddH_2_O, they were processed as described above for TEM.

### In vivo AG characterization and typing by MRI

Analysis was performed following a protocol approved by the West Virginia University Institutional Review Board (protocol number: 2207606114). For in vivo characterization of AG types and distribution, MRI examinations from 15 individuals (mean age 58.6 yr, range 30–71 yr) were retrospectively evaluated along frontal convexities. Adults who consecutively underwent MRI brain examinations on a 3T Siemens scanner as part of their standard clinical workup at a tertiary care center and whose imaging protocol included high-resolution volumetric T1, T2, and FLAIR were included. Individuals with a structural brain abnormality, prior neurosurgery, any acute intracranial process, clinical diagnosis of dementia and/or minor cognitive disorder were excluded. Subjects with a significant motion artifact limiting anatomical analysis were also excluded. MRI examinations on all subjects were performed using a 3-Tesla Magnetom Prisma MRI unit (Siemens) with a 20-channel head coil. Imaging included the following sequences: 3D T1 MPRAGE: (repetition time/time to echo [TR/TE] = 2,300/2.26 ms; 1 × 1 × 1 mm resolution; flip angle = 8); 3D T2 SPACE (TR/TE = 3,200/329 ms; 0.9 mm resolution; flip angle = 120); and 3D T2-fluid attenuated inversion recovery (FLAIR; TR/TE = 6,000/399 ms; 1 × 1 × 1 mm resolution; flip angle = 120). When available, CT images of the head were also reviewed. Image analysis was performed by an experienced board-certified neuroradiologist using FUJI Synapse picture archiving and communication system. Following coregistration of T1, T2, and FLAIR sequences, frontal convexities were divided into four sections using a method to normalize variances in the brain and calvarial dimensions. To accomplish this, a line between the anterior base of crista galli and the inner table of the calvarium over the central sulcus was equally sectioned into four segments that were used to designate frontal levels L1–L4 (i.e., anterior to posterior, respectively). Next, AG were manually counted. For each subject and at each frontal level, the number of distinct AG types was recorded. By assessing the dome location, AG were classified as Type I (sinus), Type II (stromal), Type III (diploic), Type IV (epidural), and/or Type V (subdural). Parasagittal stroma was identified as perisinus hyperintense signal on the T2-FLAIR sequence. For multilobar AG, the locations of each dome were assessed to determine the possibility of a hybrid location.

### Statistical analysis

Statistical analyses were performed using Prism 8 (GraphPad Software, Inc.) and R-studio. Data were reviewed with Q–Q plots and summary statistics, as well as scatterplots, boxplots, bar graphs, and/or barycentric plots (for % components of the AG core). Values within a person were summarized by the mean or median across the 20 granulations or as the percentage of granulations with a specific characteristic. Frontal pole AG characteristics are summarized in three patient groups: those from young persons (100 AG from five persons <50 yr at death); those from middle-aged persons (140 AG from seven persons aged 50–70 yr at death), and those from old persons (160 AG from eight persons over 70 yr at death). Similarly, AG characteristics along frontal convexities are summarized in three patient groups: those from young persons (34 AG from four persons <50 yr at death); those from middle-aged persons (50 AG from six persons aged 50–70 yr at death), and those from old persons (40 AG from five persons over 70 yr at death). DAS characteristics are summarized in these same patient groups (three DAS samples were studied from three persons per age group). Pearson correlations (r) of these summary measures with age and with each other are reported as descriptive statistics, so P values are not presented. As a guide, the two-sided cut-offs for statistical significance for the correlation of *n* = 20 pairs of values are 0.444 (alpha = 0.05), 0.561 (alpha = 0.01), and 0.602 (alpha = 0.005).

### Online supplemental material

Fig. S1 shows ultrastructural images of a unilobed AG and depicts its surface and internal morphology. Fig. S2 shows fluorescent images of a multilobed AG and depicts the relationships of fissures, sinuses, and crypts within the irregular cavernous space network. Fig. S3 shows the perisinus DAS that harbors immune cells, immune synapses, and lymphatic endothelial components. Table S1 shows a complete list of primary antibodies used in this study. Video 1 shows pedunculated AG with bridging. Video 2 shows sessile AG with fissures. Video 3 AG stroma with sequestered macrophages.

## Supplementary Material

Table S1 shows a complete list of primary antibodies used in this study.
