# Supplementary material for: Arachnoid granulations are lymphatic conduits that communicate with bone marrow and dura-arachnoid stroma
Source: J Exp Med. 2022 Dec 5;220(2):e20220618. doi: 10.1084/jem.20220618 (PMC9728136; doi:10.1084/jem.20220618)
Supplement: Table S1 — shows a complete list of primary antibodies used in this study. [file JEM_20220618_TableS1.docx]

Table S1. Key resources table

| **Antibody Type** | **Antigen** | **Catalog Number** | **Source** | **Host** |
| --- | --- | --- | --- | --- |
| **Collagen** | Pan-collagen | PA1-85324 | Thermo/Invitrogen | Rabbit |
| **Arachnoid cell** | E-cadherin | NBP2-53229 | Novus Biologicals | Mouse |
|  | EMA | ab134282 | Abcam | Mouse |
|  | PR | MA5-12658 | Thermo/Invitrogen | Mouse |
|  | Vimentin | PA1-10003 | Thermo/Invitrogen | Chicken |
| **Vascular endothelium** | Factor VIII | PA5-106877 | Thermo/Invitrogen | Mouse |
|  | PECAM (CD31) | sc-376764 | Santa Cruz Biotechnology, Inc | Mouse |
| **Lymphatic endothelium** | D2-40 (podoplanin) | MA1-83884 | Thermo/Invitrogen | Mouse |
|  | Prox1 | ab38692 | Abcam | Rabbit |
| **Erythrocyte** | CD235a | MA5-12484 | Thermo/Invitrogen | Mouse |
| **Macrophagic/monocyte** | CD68 | 14-0688-82 | Thermo/Invitrogen | Mouse |
| **Lymphocyte** | CD4 | sc-19641 | Santa Cruz Biotechnology, Inc | Mouse |
|  | CD20 | ab236434 | Abcam | Mouse |
|  | CD45 | sc-70696 | Santa Cruz Biotechnology, Inc | Mouse |
| **Plasmacyte** | CD138 | 67155 | Proteintech Group Inc | Mouse |
| **Astrocyte** | GFAP | MAB360 | Millipore/Sigma | Chicken |
| **Neutrophile** | Myeloperoxidase (MPO) | 66177-1 | Proteintech Group Inc | Mouse |
| **Neuronal element** | NeuN | ABN78 | Millipore/Sigma | Rabbit |
|  | Neurofilament | ab7794 | Abcam | Mouse |
|  | MAP2 | M4403 | Millipore/Sigma | Mouse |
|  | Synaptophysin | NBP2-80492 | Novus Biologicals | Chicken |
| **Dendritic cell** | CD11c | Ab11029 | Abcam | Mouse |
| **Mast cell** | CD117 (CKIT) | AF1356 | Novus Biologicals | Goat |
| **Adhesive protein** | Fibronectin | ab2413 | Abcam | Rabbit |
| **Proliferation marker** | Ki67-488 (conjugated) | ab197234 | Abcam | Rabbit |
| **Cell death marker** | Cleaved-caspase 3 | 9661 | Cell Signaling | Rabbit |
| **Water channel** | Aquaporin1 | PA5-53954 | Thermo/Invitrogen | Rabbit |
|  | Aquaporin4 | ab3594 | Millipore | Rabbit |
| **Cytokine/chemokine** | CCL3 | ab32609 | Abcam | Rabbit |
| **and other immune** | CCL5 | AF278 | Novus Biologicals | Goat |
| **markers** | CCL7 | NBP1-82367 | Novus Biologicals | Rabbit |
|  | CCL8 | AF790 | R&D Systems | Goat |
|  | CCL11 | MAB320 | R&D Systems | Mouse |
|  | CCL12 | AF428 | R&D Systems | Goat |
|  | CCL17 | ab182793 | Abcam | Rabbit |
|  | CCL21 | AF366 | R&D Systems | Goat |
|  | CCL25 | 25285-1-AP | Proteintech | Rabbit |
|  | CXCL1 | MAB275 | Novus Biologicals | Mouse |
|  | CXCL2 | Pa5-122058 | Thermo/Invitrogen | Rabbit |
|  | CXCL5 | AF254 | R&D Systems | Goat |
|  | CXCL9 | ab9270 | Abcam | Rabbit |
|  | CXCL10 | AF266 | R&D Systems | Goat |
|  | CXCL12/SDF-1 | MAB350 | R&D Systems | Mouse |
|  | CXCL13 | AF801 | R&D Systems | Goat |
|  | CXCL14 | ab137541 | Abcam | Rabbit |
|  | CXCL16 | NBP2-58349 | Novus Biologicals | Rabbit |
|  | CXCL17 | MAB4207 | R&D Systems | Mouse |
|  | IL-1b | ab2105 | Abcam | Rabbit |
|  | IL-2 | ab231441 | Abcam | Rabbit |
|  | IL-2R | ab9496 | Abcam | Mouse |
|  | IL-3 | sc-28342 | SantaCruz | Mouse |
|  | IL-4 | ab9622 | Abcam | Rabbit |
|  | IL-4R | MA5-23814 | Thermo | Mouse |
|  | IL-5 | 26677-1-AP | Proteintech | Rabbit |
|  | IL-6 | ab6672 | Abcam | Rabbit |
|  | IL-7 | sc-365306 | SantaCruz | Mouse |
|  | IL-8 | ab18672 | Abcam | Mouse |
|  | IL-10 | ab133575 | Abcam | Rabbit |
|  | IL-12 | ab9992 | Abcam | Goat |
|  | IL-13 | ab106732 | Abcam | Rabbit |
|  | IL-17A | ab79056 | Abcam | Rabbit |
|  | IL-23 | ab45420 | Abcam | Rabbit |
|  | Interferon-α | sc-373757 | SantaCruz | Mouse |
|  | Interferon-β | ab85803 | Abcam | Rabbit |
|  | Interferon-γ | 14-7318-81 | Thermo | Mouse |
|  | Lymphotoxin α Receptor | MAB211 | R&D Systems | Mouse |
|  | MHC-II | sc-59318 | SantaCruz | Mouse |
|  | NFκB | 14-6731-81 | Thermo/Invitrogen | Rabbit |
|  | TGF-α | ab9578 | Abcam | Mouse |
|  | TGF-β | ab215715 | Abcam | Rabbit |
|  | TNF | SC-133192 | Santa Cruz | Mouse |
|  | LTα | MAB002 | R&D Systems | Mouse |
|  | TNFR1 | ab19139 | Abcam | Rabbit |
